# Mammary epithelial cell transcriptome reveals potential roles of lncRNAs in regulating milk synthesis pathways in Jersey and Kashmiri cattle

**DOI:** 10.1186/s12864-022-08406-x

**Published:** 2022-03-04

**Authors:** Peerzada Tajamul Mumtaz, Basharat Bhat, Eveline M. Ibeagha-Awemu, Qamar Taban, Mengqi Wang, Mashooq Ahmad Dar, Shakil Ahmad Bhat, Nadeem Shabir, Riaz Ahmad Shah, Nazir A. Ganie, Dinesh Velayutham, Zulfqar ul Haq, Syed Mudasir Ahmad

**Affiliations:** 1 Division of Animal Biotechnology, Faculty of Veterinary Sciences and Animal Husbandry, Sher-e- Kashmir University of Agricultural Sciences and Technology – Kashmir, SKUAST-K, Shuhama, Jammu, 190006 India; 2 Department of Biochemistry, School of Life Sciences Jaipur National University, Jaipur, India; 3 Division of Animal Breeding and Genetics, Faculty of Veterinary Sciences and Animal Husbandry, SKUAST-K, Shuhama, Jammu, India; 4 Agriculture and Agri-Food Canada, Sherbrooke Research and Development Centre, Sherbrooke, Quebec, Canada; 5 AgriGenom laboratory Pvt. Limited, Cochin, India; 6 Division of Livestock Production and Management, SKUAST-K, Srinagar, India

**Keywords:** Cattle, Mammary epithelial cells, Lactation stages, lncRNA, RNA sequencing

## Abstract

**Background:**

Long noncoding RNAs (lncRNAs) are now proven as essential regulatory elements, playing diverse roles in many biological processes including mammary gland development. However, little is known about their roles in the bovine lactation process.

**Results:**

To identify and characterize the roles of lncRNAs in bovine lactation, high throughput RNA sequencing data from Jersey (high milk yield producer), and Kashmiri cattle (low milk yield producer) were utilized. Transcriptome data from three Kashmiri and three Jersey cattle throughout their lactation stages were utilized for differential expression analysis. At each stage (early, mid and late) three samples were taken from each breed. A total of 45 differentially expressed lncRNAs were identified between the three stages of lactation. The differentially expressed lncRNAs were found co-expressed with genes involved in the milk synthesis processes such as *GPAM*, LPL, and *ABCG2* indicating their potential regulatory effects on milk quality genes. KEGG pathways analysis of potential *cis* and *trans* target genes of differentially expressed lncRNAs indicated that 27 and 48 pathways were significantly enriched between the three stages of lactation in Kashmiri and Jersey respectively, including mTOR signaling, PI3K-Akt signaling, and RAP1 signaling pathways. These pathways are known to play key roles in lactation biology and mammary gland development.

**Conclusions:**

Expression profiles of lncRNAs across different lactation stages in Jersey and Kashmiri cattle provide a valuable resource for the study of the regulatory mechanisms involved in the lactation process as well as facilitate understanding of the role of lncRNAs in bovine lactation biology.

**Supplementary Information:**

The online version contains supplementary material available at 10.1186/s12864-022-08406-x.

## Introduction

The mammary gland is a vital organ for milk synthesis and secretion, providing essential nutrients for mammalian offspring and human nourishment. Lactation is the maternal physiological response, however, the milk yield greatly varies among cattle breeds [1, 2]. The lactation process is influenced by genetic, epigenetic, and environmental factors [3]. Elucidating regulatory mechanisms of the lactation process is not only vital for improving milk quality and production but also helps in understanding other processes related to milk synthesis. Mammary epithelial cells are the factories of milk lipids, proteins, and carbohydrates during lactation [4]. The milk-producing mammary epithelial cells (MECs) are the functional unit of mammary gland, and the proliferation of MECs is a key determinant of lactation of mammary gland [5]. Various genes and regulatory molecules are expressed at different lactation stages, which play crucial roles in the regulation of lactation [6–8]. To improve the milk production performance in cattle, deeper understanding of the molecular biology of lactating mammary glands, such as expression and regulatory mechanisms of milk related genes, including the regulatory roles of lncRNAs is essential.

Previously thought of as junk transcripts and pseudogene remnants, non-coding RNA molecules including lncRNA have emerged as essential components of cellular activity, regulating a plethora of functions within multicellular organisms [9]. LncRNAs are defined as transcripts ≥200 bp in length and without the potential to code for a protein. They are often polyadenylated and usually exhibit low expression levels and poor sequence conservation [10–13]. LncRNAs activate or repress genes at multiple levels (i.e., DNA, RNA and proteins) by acting as guide, signaling, scaffolding or decoy molecules [14]. It has been shown that lncRNAs play important roles in certain tissues as well as have species-specific expression [15], but some multifunctional lncRNAs have also been shown to display broad and conservative expression profiles [16, 17]. In mammals, lncRNAs are known to play roles in various biological processes including growth, reproduction and health [18, 19]. It is also well established that lncRNAs are involved in the development of the mammary gland and diseases like breast cancer [20–22]. In bovine, the lncRNA repertoire in several tissues including the mammary gland [23–28], milk exosomes [29], skeletal muscle/adipose tissues [30, 31] gastrointestinal tract tissues [32, 33] and sperm/testis [34, 35] has been characterized. Increasing evidence supports the notion that lncRNAs are associated with developmental, metabolic and immunological regulation, as well as adaptations and phenotypic variation of complex traits in domestic animals [26, 36, 37]. However, the role of lncRNA in the bovine milk production and mammary gland processes is less clear.

The overall objective of this study was to systematically identify the profiles of differentially expressed lncRNAs during different stages of lactation in cattle through high-throughput RNA sequencing. We hoped that by studying the relationship between differentially expressed (DE) lncRNAs and milk quality and yield related genes (mRNAs), our study would shed light on the complex mechanisms underlying the milk production.

## Materials and methods

### Experimental animals and sampling

The experimental design was the same to that of our earlier study [38], RNA-Sequencing data were download from GSE107366. Briefly, the data from three healthy cows of each Kashmiri and Jersey breed in their 2nd/3rd parity/lactation were utilized. All animals were of same age and milk yield was also same. Animals were maintained at the University dairy farm, Mountain Livestock Research Institute, Share-Kashmir University of Agricultural Sciences and Technology-Kashmir, India. The animals were stall fed individually, offered complete feeds based on oats hay (30 parts), sorghum (30 parts) and concentrate mixture (40 parts) to meet their nutrient requirements as per ICAR, India (2013). The concentrate mixture consisted of maize (20%), wheat bran (25%), deoiled rice bran (15%), mustard oilcake (15%), cotton seed cake (16%), molasses (5%), mineral mixture (2%), salt (1%) and urea (1%). Fresh milk samples (1.5 L/cow) were collected in the morning aseptically by hand milking the four quarters of the cows at 15 (D15), 90 (D90) and 250 (D250) days in milk, representing early, mid and late lactation stages, respectively. The milk production was recorded in kgs. and values are significantly comparable between the animals [38].

### Isolation of milk epithelial cells

Milk epithelial cells (MECs) were isolated from freshly collected milk following the protocol [4] with some modifications as reported in our previous study [38]. Briefly, milk sample (1.5 L) was aliquoted into 250 ml centrifuge tubes, and 100 ml of 4 °C diethylpyrocarbonate (DEPC) treated phosphate buffered saline (PBS) added. Samples were centrifuged at 2800 x g at 4 °C for 2 min and the fat and skimmed milk were removed. The pellet and the remaining supernatant fraction (1 ml) were mixed with 800 μl of DEPC–PBS and transferred into a 2 ml tube. After adding 200 μl EDTA (0.5 M pH 8.0, 4 °C), the samples were centrifuged at 14,000 g for 1 min at 4 °C. The supernatant was discarded, and the pellets resuspended in 200 μl cold (4 °C) DEPC–PBS. Solutions containing pellets from the same cow were pulled together and the suspension centrifuged at 5100 x g for 5 min at 4 °C. Thereafter, the supernatant was discarded, and the pellet resuspended in 1.25 ml cold (4 °C) PBS containing 1% bovine serum albumin (Sigma, USA). For the separation of MEC from other cell types, MEC specific anti-cytokeratin peptide 18 antibodies (Clone KS-B17.2, Sigma–Aldrich, USA) coated beads (Dynabeads Pan Mouse IgG, Invitrogen) were used. The Purified MECs were checked for possible contamination with other cell types by quantification of the expression of marker genes for various milk somatic cell types as described by Bhat et al. [38].

### RNA extraction and library preparation

RNA extraction was accomplished with Trizol Reagent (Ambion, USA) according to the manufacturer’s instructions. Absorbance (A) of RNA samples was measured at 260 and 280 nm using the spectrophotometer (Thermo Scientific, USA). The A260 was used to estimate the RNA concentration. Moreover, the quality and integrity were assessed with the Agilent 2100 Bioanalyzer (Agilent, USA). The RNA integrity number (RIN) value of the samples used for library preparation was ≥8. IlluminaTruSeq Stranded mRNA Sample Prep kit (Illumina, USA) was used to generate cDNA libraries from 4μg total RNA according to the manufacturer’s recommendations. Poly-T oligo-attached magnetic beads were used to purify poly-A containing mRNA and lncRNAs molecules followed by fragmentation into small pieces using divalent cations under elevated temperature. First strand cDNA was synthesized using reverse transcriptase and random primers followed by second strand cDNA synthesis using DNA Polymerase I and RNase H. After adenylation of 3′ ends of DNA fragments, hybridization was initiated by ligating Illumina PE adapter and index. cDNA fragments (200 bp) were generated and were selectively enriched to construct the final sequencing library using Illumina PCR Primer Cocktail. The sequencing was performed at SciGenome Lab (Cochin, India) using Illumina Hiseq 2500. The data was retrieved from our submitted NCBI SRA database (accession ID. SRR6324372/GSE107366).

### Sequence data quality control, mapping and identification of lncRNAs

The raw reads were subjected to quality control using the FASTQC program v0.11.9 [39] and cleaned using cutadapt tool version 3.2 [40] and sickle tool [41]. All clean reads were mapped to the *Bos taurus* reference genome assembly ARS-UCD1.2 using HISAT2 v2.2.1 [42]. The final transcript sets were compared with known genes annotated by Ensemble Release 94. Known protein-coding transcripts and small noncoding RNA transcripts (e.g. sRNAs, tRNA, rRNA, etc.) were removed while annotated lncRNA transcripts were retained using homologous sequence similarity search with BLAST program [43, 44]. Next, the transcripts with length of < 200 bp were removed. The remaining transcripts that did not overlap with any known annotation, localized in intronic, antisense or intergenic regions were assessed for their protein coding potential using four independent algorithms, CNCI (coding-non-coding index) [45], PLEK (predictor of long non-coding RNAs and messenger RNAs based on an improved k-mer scheme) [46], CPAT (coding potential assessment tool) [47], and Pfam (database providing alignments and hidden Markov models for protein domains) [48, 49].

CPAT recognizes coding and noncoding transcripts from a large pool of candidates using a logistic regression model built with four sequence features: open reading frame size, open reading frame coverage, Fickett TESTCODE statistics and hexamer usage bias. CPAT coding probability score ranges from 0 to 1, and the optimum cut-off for protein coding probability varies depending on the species being analyzed. In order to extract potential noncoding transcripts with a high reliability from our dataset, we selected a stringent threshold for the CPAT probability with a score < 0.02 as ncRNA [12]. The transcripts with a score below the selected thresholds were classified to possess an ambiguous coding potential. Furthermore, Pfam Scan (v1.3) was employed to identify occurrences of any known protein family domain documented in the Pfam database. CNCI software is a signature tool that effectively distinguishes between protein-coding and non-coding sequences based on their intrinsic sequence composition i.e., by profiling adjoining nucleotide triplets. The alignment-free tool, PLEK, uses a computational pipeline based on an improved k-mer scheme and a support vector machine algorithm to distinguish lncRNAs from messenger RNAs (mRNAs). This tool has > 90% accuracy when compared with other currently available tools [50].

### Differential expression analysis

The expression levels of lncRNAs between any two stages of lactation, were measured as fragments per kilobase of exon per million fragments mapped (FPKM) using Cufflinks v2.1.1 package [51, 52]. Differentially expressed lncRNA was screened based on FDR corrected *p*-value < 0.05 and absolute log2(fold change) > 1.

### Expression correlation analysis between lncRNAs and genes related to milk quality and yield traits

To identify correlation between DE lncRNA and mRNAs, Pearson correlation test was performed to calculate the co-expression coefficient between lncRNA expression data and mRNAs [53]. Two-way analysis of variance (ANOVA) was used to evaluate the statistical significance of comparisons within the lactation stages using R program v4.0.0. *P*-values (two-sided) were adjusted for FDR due to multiple testing correction [54] and FDR < 0.05 was defined as statistically significant. A lncRNA-mRNA pair was considered co-expressed if it had a significant correlation value at FDR < 0.05.

### Target gene prediction and functional analysis

For the identification of lncRNA trans-target genes, Spearman correlation was calculated between DE-lncRNAs and DE-mRNA using COR.TEST function in R [53]. Then interactions of DE-lncRNAs and DE-mRNAs with r values ≥0.9 and *P* < 0.05 were selected as trans-target genes. The *cis* role of lncRNAs was expounded as those influencing neighboring target genes [55]. For each DE lncRNA, the nearest upstream and downstream (within 100 kb) protein-coding neighbors were identified as their potential *cis*-regulatory targets. The *trans* role alludes to the impact of lncRNA on mRNA at the expression level. The expressed correlations between lncRNAs and coding genes were calculated using the Pearson method with *p*-value < 0.05. To understand the biological implication of the target genes we performed GO and KEGG enrichment analysis utilizing KOBAS server version 3 [56, 57]. The GO terms were categorized into biological processes (BP), cellular components (CC), and molecular functions (MF). GO terms and pathways with a *p*-value less than 0.05 were considered as significantly enriched.

### Quantitative real time PCR (qRT-PCR) validation of lncRNAs

To validate the repeatability and reproducibility of the RNA-Seq data, quantitative real-time PCR was performed on 10 randomly selected lncRNAs (including 5 up- and 5 down-regulated) using the same total RNA that was used to perform the RNA sequencing. Primers for qRT-PCR were designed using Primer 5.0 software (http://www.premierbiosoft.com/primerdesign/) and their specificity and complementarity were assessed using NCBI BLAST algorithm (Supplementary File 1). The complementary DNA (cDNA) was synthesized from 0.5 μg of total RNA with the Revert Aid First Strand cDNA Synthesis Kit (Thermo Scientific, USA). Real-time PCR reaction mix was composed of 10 μL SYBR Green PCR Master Mix (Roche, Germany), 0.5 μL cDNA, 0.3 μL (10 mM) forward and reverse primers and 9.2 μL nuclease free water to a final volume of 20 μL. All aliquots were then amplified by 40 cycles of denaturation at 95 °C for 5 min, annealing at 60 °C for 15 s and extension at 72 °C for 15 s. The qRT-PCRs were carried out in triplicates. The expression levels of the selected lncRNAs were normalized against two housekeeping genes, *GAPDH* and *UXT.* The relative quantification of the potential lncRNAs was determined using the 2^-ΔΔCt^ method [58].

## Results

### Sequencing results and quality control

Transcriptome sequencing of 18 cDNA libraries (three at each stage of lactation per breed) generated a total of 305, 321, and 241 million raw reads for Jersey and 248, 237 and, 302 million raw reads for Kashmiri cattle at early (D15), mid (D90) and late (D250) lactation stages, respectively. Out of these, 302 million (D15); 315 million (D90) and 217 million (D250) reads for Jersey and 235 million (D15); 235 million (D90) and 292 million (D250) reads for Kashmiri cattle passed quality control. On average, 95.8% raw reads from each library passed the quality check. Alignment of reads to the bovine reference genome ARS-UCD1.2 yielded a mean of 90.1% (Kashmiri) and 90.9% (Jersey) unique alignments to the reference genome while 9.9% (Kashmiri) and 9.1% (Jersey) reads aligned to multiple positions or did not align at all and were discarded (Table 1).

**Table 1:** Read alignment summary

Sample No.	Total reads	QC passed reads	QC Passed %age	Aligned reads	Aligned Read %age	Unaligned Reads	Unaligned Reads %age
JERSEY-390-1	96,249,540	94,744,250	98.44%	85,869,238	90.63%	8,875,012	9.37%
JERSEY − 390-2	92,517,372	91,969,506	99.41%	84,113,665	91.46%	7,855,841	8.54%
JERSEY −390-3	116,401,530	115,758,078	99.45%	106,605,825	92.09%	9,152,253	7.91%
JERSEY − 447-1	91,214,046	89,570,316	98.20%	79,097,456	88.31%	10,472,860	11.69%
JERSEY −477-2	93,235,128	89,955,200	96.48%	81,828,632	90.97%	8,126,568	9.03%
JERSEY −477-3	136,828,434	135,768,664	99.23%	125,418,364	92.38%	10,350,300	7.62%
JERSEY −90-1	90,605,106	76,773,936	84.73%	69,093,481	90.00%	7,680,455	10.00%
JERSEY 90-2	73,121,688	69,887,565	95.57%	63,996,224	91.57%	5,891,341	9.20%
JERSEY −90-3	77,649,922	70,905,710	91.31%	65,212,246	91.97%	5,693,464	8.03%
KASHMIRI-BL1-1	82,462,774	72,319,896	87.70%	62,308,532	86.16%	10,011,364	13.84%
KASHMIRI -BL1-2	73,887,336	73,339,212	99.26%	67,574,186	92.14%	5,765,026	7.86%
KASHMIRI -BL-1-3	92,098,428	89,792,054	97.49%	84,924,806	94.58%	4,867,248	5.42%
KASHMIRI -ML2-1	81,811,960	71,855,586	87.83%	59,079,820	82.22%	12,775,766	17.78%
KASHMIRI -ML2-2	68,425,836	66,722,732	97.51%	59,373,810	88.99%	7,348,922	11.01%
KASHMIRI -ML-2-3	87,720,824	86,711,472	98.85%	80,073,868	92.35%	6,637,604	7.65%
KASHMIRI -SL3-1	104,571,152	102,595,298	98.11%	90,594,073	88.30%	12,001,225	11.70%
KASHMIRI -SL3-2	76,974,734	76,313,042	99.14%	70,145,228	91.92%	6,167,814	8.08%
KASHMIRI -SL-3-3	120,888,074	113,697,196	94.05%	105,425,162	92.72%	8,272,034	7.28%

### Identification and characterization of lncRNAs in milk derived bovine mammary epithelial

**Fig. 1:**
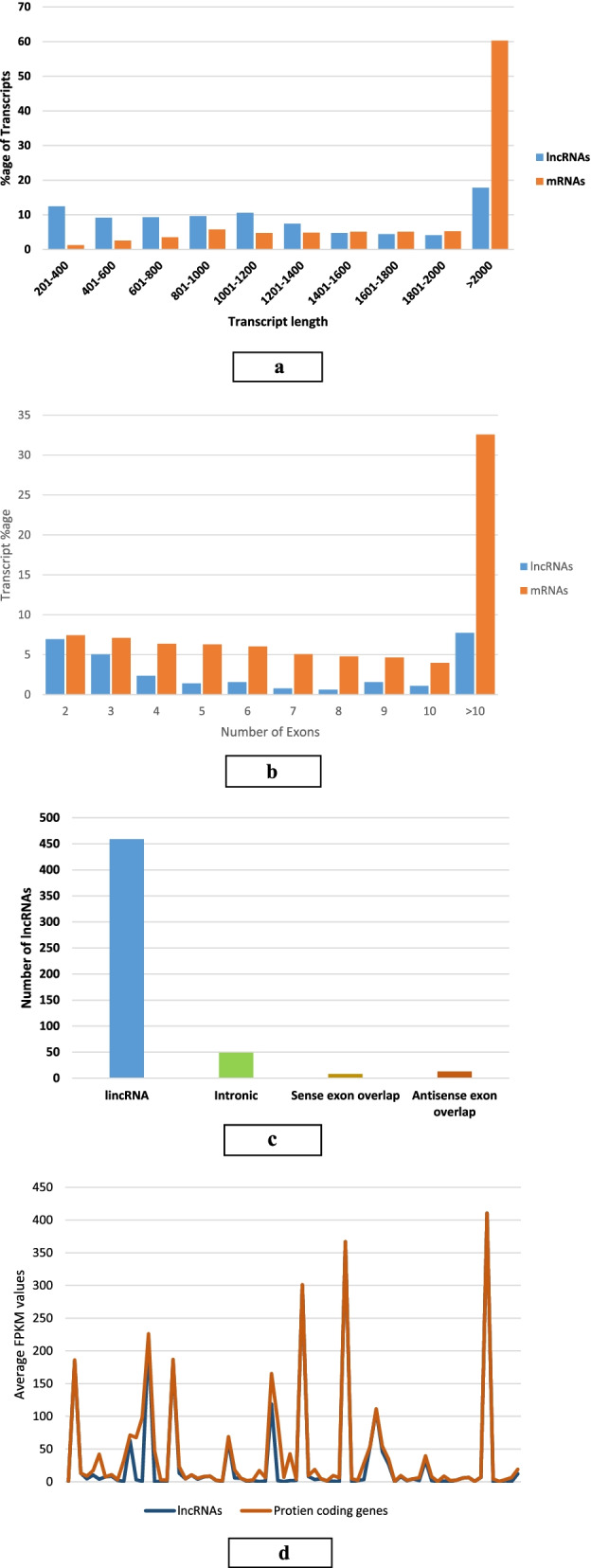
Features of lncRNAs compared with protein coding genes (mRNAs). **A** Length distribution of lncRNAs compared with mRNAs. **B** Exon number distribution of lncRNA transcripts compared with mRNAs. **C** Classification of identified lncRNAs on the basis of genomic location and orientation. **D** Comparison of average expression values of lncRNAs and mRNAs

To predict lncRNA with greater certainty, four independent algorithms, CPAT, CNCI, PFAM and PLEK, were used. A total of 549 unique lncRNAs were predicted by the four independent algorithms (Supplementary File 2). The transcript length of identified lncRNAs ranged from 206 to 15,071) nucleotides, with ~ 44% of lncRNAs shorter than 1000 nucleotides (Fig. 1a, Supplementary File 2). Exon number of lncRNAs ranged from 2 to 43. Most lncRNAs had two exons (57.1%), followed by three exons (7.6%) and about 35.3% of lncRNAs had more than four exons (Fig. 1b). Characterization according to genomic location indicated that most lncRNAs are located in intergenic regions (459 lncRNAs), whereas 49, 13 and 8 lncRNAs are intronic, antisense or sense-overlapping lncRNAs, respectively (Fig. 1c).

To determine whether our set of identified lncRNAs possesses similar characteristics as mRNA, we compared the lncRNA expression data with mRNA expression data on the same samples. Results showed that, the lncRNAs were generally less expressed as compared to protein-coding mRNAs (Fig. 1d).

### DE lncRNAs between lactation stages in Kashmiri and Jersey cattle

**Fig. 2:**
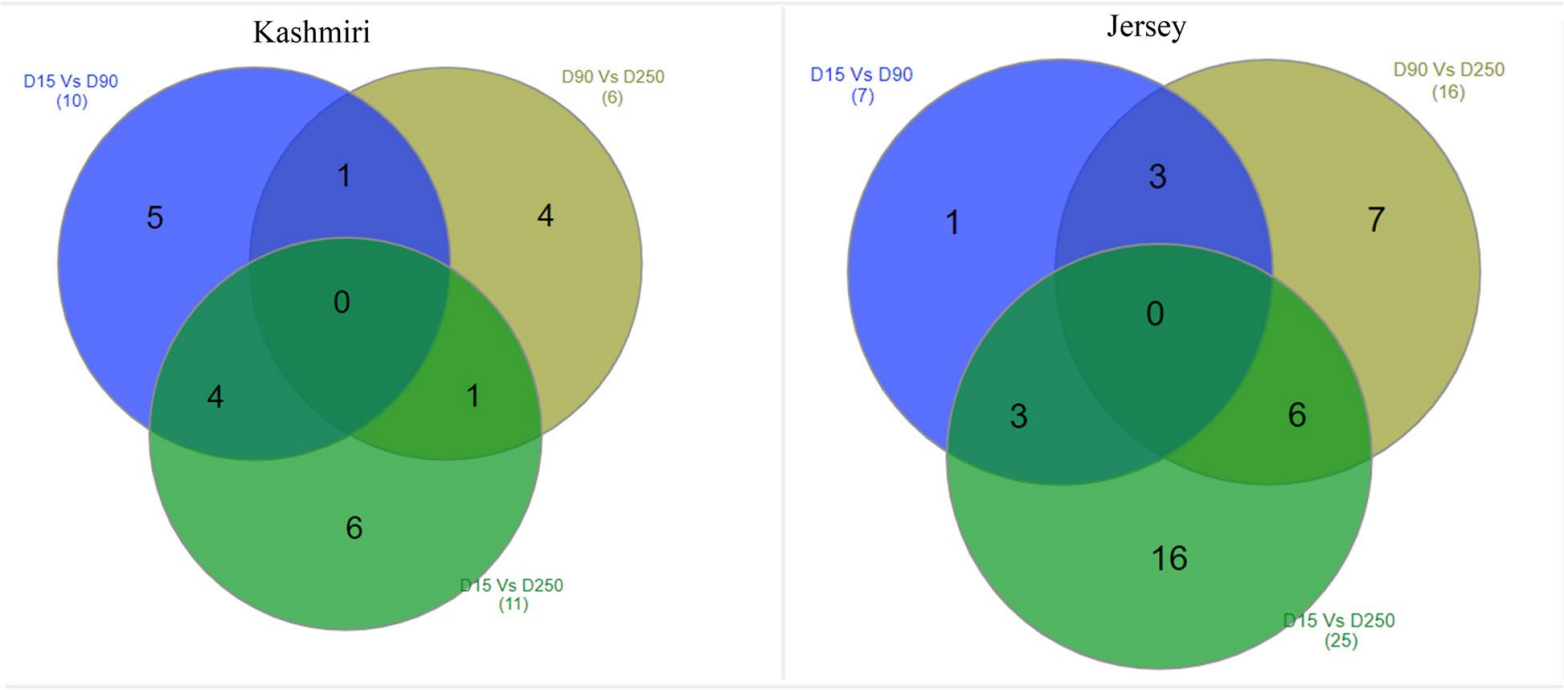
Significantly differentially expressed lncRNAs between D15 vs D90, D15 vs D250 and D90 vs D250 in Kashmiri and Jersey cattle

A total of 10, 6 and 11 lncRNAs were DE (FDR < 0.05) between D15 vs D90, D90 vs D250, and D15 vs D250, respectively in Kashmiri cattle (Fig. 2, Supplementary File 3). Likewise, 7, 16 and 25 lncRNAs were DE (FDR < 0.05) between D15 vs D90, D90 vs D250, and D15 vs D250, respectively in Jersey cattle (Fig. 2, Supplementary File 3). Furthermore, there were 5 common lncRNAs [*XLOC_013668* (D15 = 466.515; D90 = 93.3322; D250 = 32.1239), *XLOC_013477*(62.1684; 306.217; 131.523), *XLOC_021768*(1.8902; 7.13654; 8.20963), *XLOC_000865*(1.19188; 2.83428; 2.91069), *XLOC_003635*(0.366344; 0.883615; 0.916974)] and 12 [*XLOC_015754*(9.73475; 1.73083; 5.26697), *XLOC_009502*(0.415893; 0.0868364; 0.912991), *XLOC_003704*(1.3756; 0.300084; 1.71051), *XLOC_026943*(19.1323; 38.3812; 198.936), *XLOC_002387*(0.707159; 0.492698; 2.71744), *XLOC_002110* (0.0720901;0.336411; 3.08172), *XLOC_005292*(0.409118; 0.770406; 15.3821), *XLOC_015932*(1.12151; 1.52169; 7.63437), *XLOC_025341*(0.0476; 0.08765; 0.51983), *XLOC_020132*(96.1583; 28.32; 11.9587), *XLOC_015281*(8.484; 1.857; 2.607), *XLOC_007175*(0.04296; 1.4446; 3.41824), showing expression in all the 3 comparison groups of Kashmiri and Jersey cattle respectively.

### Functional prediction of the roles of differentially expressed lncRNAs

To explore the functional role of DE lncRNAs, we identified their *ci*s and *trans* target genes. A total of 459 (84 in Jersey and 375 in Kashmiri) potential *cis* and 8877 (6798 in Jersey and 2079 in Kashmiri) *trans* target genes were identified for 48 (in Jersey) and 27 (in Kashmiri) DE lncRNAs. To obtain an insight into the plausible associated functions of lncRNAs at different stages of lactation, the potential target genes of DE lncRNAs in three comparisons (D15 vs. D90, D90 vs. D250, and D15 vs. D250) were subjected to GO and pathway enrichment analyses. In Kashmiri cattle, target mRNAs of DE lncRNAs were enriched in 499 GO terms. Among them, 252 were categorized as *biological processes* (BP), 63 *molecular functions* (MF), and 184 *cellular component* (CC) terms. Whereas in Jersey cattle, 507 enriched GO terms were found. Among them, 245 were BP, 64 were MF and 198 were CC. The top three BP GO terms for Kashmiri cattle for each pair of comparison groups are *regulation of lipid transport*, *metabolic process*, and *cholesterol homeostasis* (D15 vs D90); *cellular macromolecule metabolic process*, *lipid homeostasis* and *intracellular protein trans-membrane transport* (D90 vs D250); and *regulation of cellular metabolic process*, *secretion by cell and response to stress* (D15 vs D250) (Supplementary Files 4 and 5). Whereas in Jersey cattle top enriched BP terms are *response to lipid*, *DNA biosynthetic process*, and *fatty acid homeostasis* between D15 vs D90. *Regulation of macromolecule biosynthetic process*, *vesicle targeting*, and *RNA processing* are enriched terms in D90 vs D250. *Golgi vesicle transport*, *regulation of cholesterol transport* and *regulation of immune system process* are found enriched between D15 vs D250. Interestingly, several BP terms related to lactation, including *regulation of gastrulation*, *fatty acid homeostasis*, *regulation of cholesterol transport* and *golgi vesicle transport* were identified.

Results of pathways analysis indicated that 10, 11, and 19 pathways were significantly enriched in Jersey cattle for D15 vs D90, D15 vs D250 and D90 vs D250 comparisons, respectively (Supplementary Files 4 and 5). Similarly, 10, 6 and 10 pathways were significantly enriched in Kashmiri cattle for D15 vs D90, D15 vs D250 and D90 vs D250 comparisons, respectively. *Homologous recombination*, *sphingolipid metabolism* and *DNA replication*; *metabolic pathways*, *carbon metabolism* and *biosynthesis of amino acids*; *DNA replication*, *mismatch repair* and *TLR signaling pathway* were the top pathways for D15 vs D90, D15 vs D250 and D90 vs D250 comparison groups in Jersey cattle. While *mTOR signaling pathway*, *WNT signaling pathway* and *PI3K-Akt signaling pathway*; col*lecting duct acid secretion*, *fanconi anemia pathway*, *N-Glycan biosynthesis* and *adherens junction* were the top enriched pathways for D15 vs D90, D15 vs D250 and D90 vs D250 comparison groups in Kashmiri cattle.

### Expression correlation network of lncRNAs with candidate genes related to milk quality and yield traits in Jersey and Kashmiri cattle

**Fig. 3:**
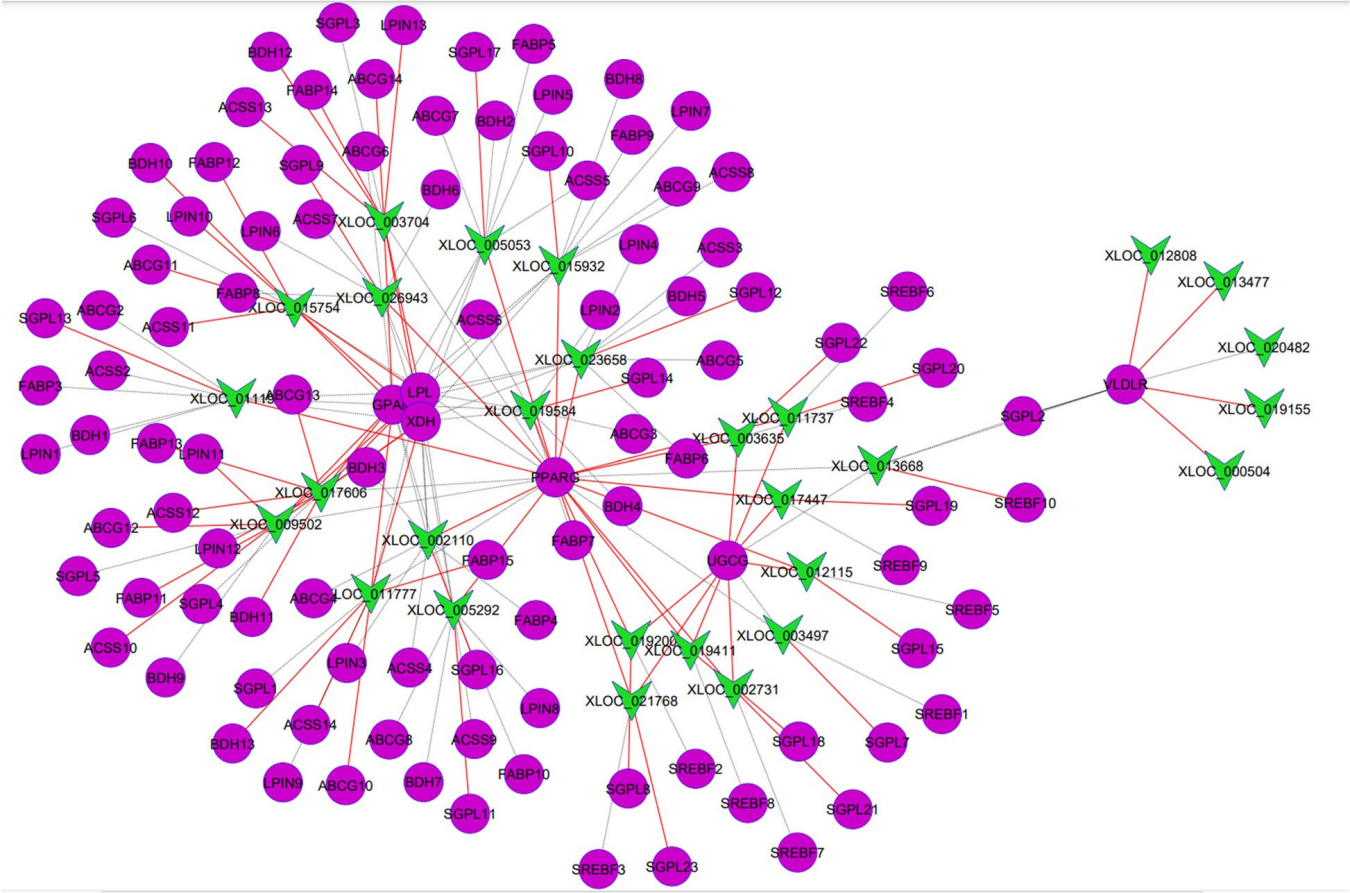
LncRNAs and milk quality and yield related genes co-expression network. The green triangles represent differentially expressed lncRNAs while pink spheres represent candidate genes for milk quality and yield traits. Red lines represent positive correlations while black dotted lines represent negative correlations

To further explore the potential regulatory mechanism of the DE lncRNAs, their relationship with protein-coding genes with roles in milk quality and yield traits was explored through correlation analysis. The results of lncRNA and coding genes correlation analysis are shown in Supplementary File 6. 3768 (Jersey) and 4048 (Kashmiri) significant correlations (FDR < 0.05) were found between lncRNAs and their potential target mRNAs. Furthermore, 360 significant correlations were found between DE lncRNAs and 46 candidate genes for milk quality and yield traits (179 and 189 in Jersey and Kashmiri cattle, respectively) (Fig. 3). Among the 179 correlations in Jersey, 104 were positive and 65 were negative correlations while of the 189 correlations found in Kashmiri cattle, 117 were negative and 72 were positive correlations. Interestingly, we found that 13 lncRNAs correlated with *LALBA* in Jersey cattle while no lncRNA correlated with this gene in Kashmiri. In addition, *XLOC_011777* correlated positively with *GPAM* and *ABCG2* genes in Jersey while the correlation of *XLOC_011777* was negative with both genes in Kashmiri cattle. Interestingly, the 5 commonly expressing lncRNAs in Kashmiri cattle were showing correlation with *UGCG, VLDLR, SREBF1, PPARG, SGPL1* where as the 12 commonly expressing lncRNAs were showing expression correlation with *GPAM, ABCG2, SCAP, FASN, SPTLC1, UGCG, BDH1, LALBA, SLC2A8, LPL, CSNK1, NOS2,* and *MFGE8*. All these genes are promising candidate genes and central for milk protein and fat synthesis regulation hence the yield.

### Verification of lncRNA expression profiles using qRT-PCR

**Fig. 4:**
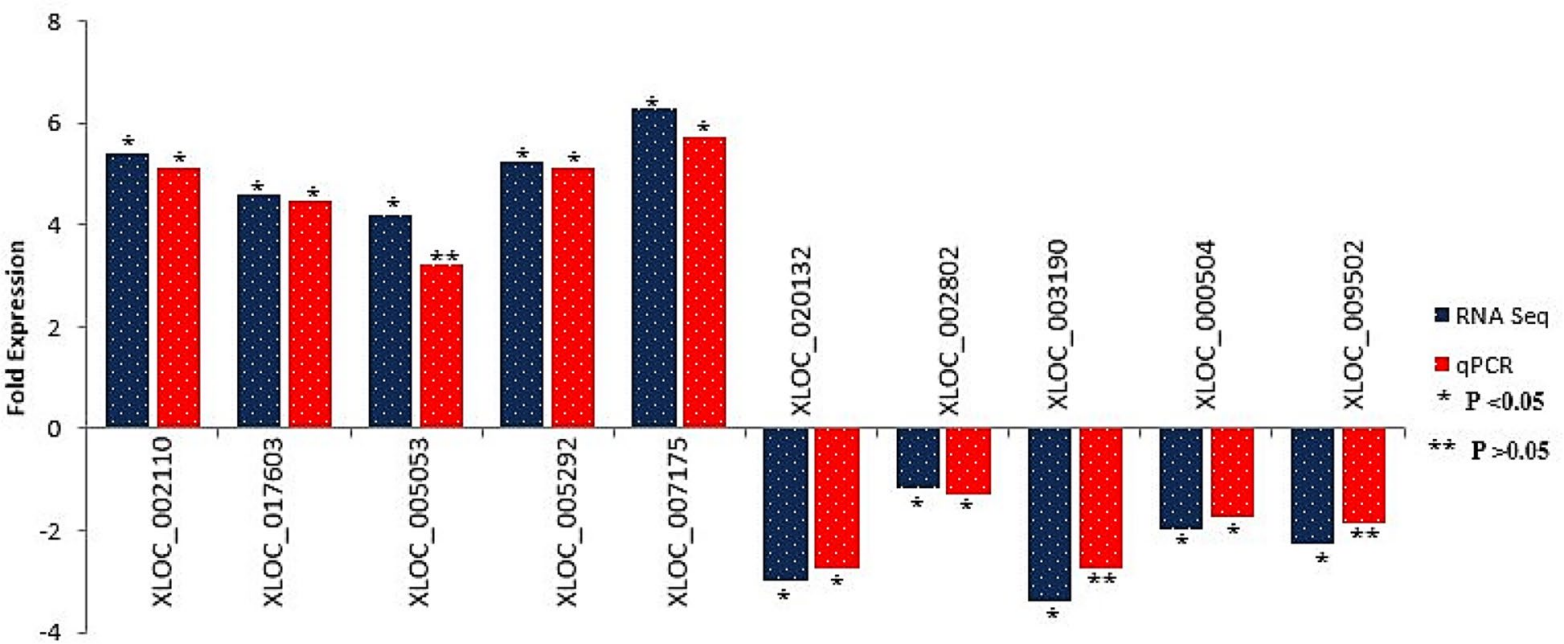
Real time quantitative PCR (qPCR) validation of high throughput sequencing data (RAN-Seq). Validation of 10 randomly selected significantly differentially expressed lncRNAs. The y-axis represents the log2 fold change of lncRNA expression; x-axis shows the lncRNA IDs. Blue and Red bars depict the RNA-Seq and qPCR results, respectively

To confirm the RNA-Seq data, the expression of 10 randomly selected lncRNAs (5 up- and 5 down-regulated) was examined by qRT-PCR. The expression patterns of lncRNAs showed a similar trend between the methods of RNA-Seq and qRT-PCR (Fig. 4). Pearson correlation coefficient between RNA-Seq data and qRT-PCR data was 0.97, indicating that the RNA-Seq data was highly correlated with that of the qRT-PCR.

## Discussion

Progressively, studies are unraveling the roles of non-coding RNA molecules in biological processes, but their roles are still classical in the area. The mammary epithelial cells are the primary cells involved in synthesis and secretion of milk and proteins in it. Lactation process is influenced by various molecule, including lncRNAs. Until now, very little is known about the bovine lncRNA transcriptome in mammary epithelial cells. In this study, we conducted a preliminary investigation of lncRNA expression profiles in mammary epithelial cells of Jersey and Kashmiri cows to assess the potential of lncRNAs as regulators of milk quality and yield during the three stages of lactation curve. Consequently, the present work provides an important resource of lncRNA repertoire in mammary epithelial cells for future studies.

The identification and characterization of lncRNAs, particularly in the mammary gland, is limited compared with lncRNAs in humans and other model organisms [59–61]. In bovine mammary gland, the main focus has been on genes and miRNAs rather than on lncRNAs [62–64]. Identification of breed-specific, milk related lncRNAs along with their expression pattern during the different stages of lactation could provide an insight into their contribution to the inter-breed variation in milk yield and could be targeted to increase the milk yield and consequently improve the profitability of the farm system in future. In addition, knowledge of these special genetic elements between breeds shall be useful to develop accurate genomic prediction equations that can operate effectively across breeds. In the present study, we identified 549 putative lncRNAs with high confidence across three lactation stages in two bovine breeds with differing milk production ability. We classified predicted lncRNAs into categories based on their genomic location to understand their functional spectrum as the relative position between a lncRNA and its neighboring protein-coding genes is a key determinant of their regulatory relationship [65]. To our knowledge, this is the first report to systematically identify lncRNAs from RNA-seq data during different stages of the bovine lactation. The identified lncRNAs have fewer exons, shorter transcript lengths, and lower expression levels in comparison with known protein-coding transcripts, which are in agreement with earlier report [66].

In total 27 and 48 lncRNAs were differentially expressed in pairwise comparisons in Kashmiri and Jersey, respectively. These lncRNAs may have specific biological roles in bovine mammary gland during the lactation cycle. As it has been demonstrated that lncRNAs are key players in tissue physiology and organogenesis [27, 67–70]. We also identified 5 and 12 DE-lncRNAs expressing in all the 3 lactation stages of Kashmiri and Jersey cattle respectively. These DE-lncRNAs were correlated with a network of genes essential for coordinating milk synthesis and secretion. Compared with Kashmiri cattle, the mammary tissue transcriptome of Jersey cattle had a completely different rank of expressing lncRNAs in terms of abundance. Recently, a study on the bovine mammary gland identified 36 lincRNAs (long intergenic ncRNAs) located in milk related quantitative trait loci (QTL), suggesting their association with milk quality and production [28]. Another study characterized lncRNAs in mammary gland tissues of cows at mid lactation and identified lncRNAs with potential roles in mammary gland functions [27]. The identification of lncRNAs associated with the development of mammary gland and lactation will contribute in selecting decisions to further improve productivity and healthy breeding policies of cattle.

It is well established that an array of genes is involved, directly or indirectly with growth and development of the mammary gland as well as initiation and maintenance of the lactation cycle [71, 72]. LncRNAs can regulate the expression of their neighboring genes (known as in *cis*) as well as distant genes (known as in *trans*) [73]. In this study, bioinformatics analysis of putative lncRNAs target genes suggest roles in pathways such as *MAPK*, *PI3K-Akt*, *NF-kappa B*, *mTOR*, T cell receptor signaling, Hedgehog (SHH) signaling pathway, Glucagon signaling pathway, *AMPK* signaling pathway, Insulin signaling pathway, and Toll-like receptor signaling pathways. Key roles for these pathways in mammary gland development and lactation have been reported [74–77]. The SHH signaling is an essential pathway and is involved in mammary gland development [78]. Moreover, the Glucagon regulates the mammary gland development and lactation through activating GPCR [79]. *AMPK* plays an essential role in cellular energy sensing [80] and mTOR activation [81]. This signaling pathway is found involved in regulating the effect of glucose supply and utilization in the lactating mammary gland [82]. Insulin signaling pathway is an important regulator of milk synthesis and secretion in the lactating animals [83]. Also, lncRNAs like XLOC_011777, XLOC_019584, XLOC_011194, and XLOC_003497, were found to interact with some lactation-related candidate genes like *LALBA*, *LPL*, *GPAM*, *SREB1* and *LIPIN1* genes. These genes have an important role in the biosynthesis of milk fat, protein, and lactose [84, 85]. In human, cow and mouse lactation cycles, *LALBA* expression levels were found to be similar to that of other milk protein genes (146, 68 and 96% expression relative to β-casein) and levels of expression of both these milk protein genes increased dramatically with onset of lactation [86]. During lactation lipoprotein lipase (LPL) is elevated in mammary tissue and depressed in adipose tissue to redirect lipids for incorporation into milk fat [87]. While activation of *SREB1*, together with *THRSP* and *ESRRA* via the concomitant decrease in progesterone concentration and increase prolactin signaling, is most likely central for milk lipid synthesis regulation in the human mammary gland [88].

Comprehensive analysis of lncRNAs and mRNAs expression profiles gives a better understanding of the biological functions of DE lncRNAs. Therefore, we performed correlation analysis of the DE lncRNAs and protein coding genes, and identified significant correlations between the transcripts in both breeds. Narrowing down the analysis to candidate genes related to milk quality and yield traits, we found that 34 lncRNAs correlated with candidate genes for milk quality and yield traits suggesting potential roles in lactation. Interestingly, most of the lncRNAs correlated positively with candidate genes related to milk quality and yield traits in Jersey compared to Kashmiri cattle where they were mostly negatively correlated, which could be one of the mechanisms responsible for the differential milking performance between the two breeds. Importantly, some of the described lncRNAs might target mRNAs, which have important roles in the mammary gland throughout the lactation cycle. For example, lncRNA *XLOC_002110* is expressed at D90 (peak lactation) stage in Jersey only and shows correlation with *SLC2A8* gene. *SLC2A8* is a member of the transporter superfamily with predominant roles in the active transport of glucose across the plasma membrane [89]. Higher expression of *SLC2A8* has been reported during mid-lactation (D90) in Jersey cows (high yielding) [39], suggesting important roles during the peak stage of lactation. Glucose uptake by mammary epithelial cells is an important step in milk synthesis during lactation, and hence directly influences the milk yield [90]. Therefore, *XLOC_002110* might be involved in the lactation process through regulation of the expression of *SLC2A8*. Based on these results, it is clear that target genes of the putative lncRNAs like *XLOC_002110*, *XLOC_019584*, *XLOC_011194*, *XLOC_005773* and *XLOC_003497* could be involved in bovine mammary gland development and their roles need to be confirmed. Based on our results and data from recent studies, lncRNAs as core regulatory elements have significant roles in the physiology of the lactation cycle and in the development of the mammary gland in cattle.

## Conclusion

In this study, 549 putative lncRNA transcripts were found at three stages of lactation in Jersey and Kashmiri cattle breeds. A total of 27 and 48 lncRNAs were DE between at least one comparison pair in Kashmiri and Jersey cattle, respectively. Target genes of DE lncRNAs were enriched in pathways (*MAPK*, *PI3K-Akt*, *NF-kappa B*, *mTOR*, T cell receptor signaling and Toll-like receptor signaling pathways) with roles in lactation and mammary gland development. Expression correlation analysis reveals that lncRNAs like *XLOC_002110*, *XLOC_019584*, *XLOC_011194*, *XLOC_005773* and *XLOC_003497* might be important regulators of candidate genes for milk quality and yield traits in cattle. Compared to Kashmiri cattle a strong correlation between DE lncRNAs and milk candidate genes was found in Jersey, which could explain the differences in milking performance between the two breeds. This study mapped the expression profiles of lncRNAs across lactation stages and their relationships with candidate genes related to milk quality and yield traits in Jersey and Kashmiri cattle. This study therefore provides a valuable resource for the study of lncRNA roles in lactation biology. However, better understanding of the molecular mechanisms involving lncRNA functions in milk synthesis of the animals is of prime importance.

## Supplementary Information

**Additional file 1.**

**Additional file 2.**

**Additional file 3.**

**Additional file 4.**

**Additional file 5.**

**Additional file 6.**

## Data Availability

Publicly available datasets were analyzed in this study. This data can be found here: https://www.ncbi.nlm.nih.gov/sra/SRR6324372

## Abbreviations

LncRNAs: Long non-coding Ribonucleic acids
MEC: Mammary epithelial cells
DE: Differentially expressed
GPAM: Glycerol-3-phosphate acyltransferase 1, mitochondrial
LPL: Lipoprotein lipase
ABCG2: ATP-binding cassette super-family G member 2
RNA: Ribonucleic acid
DNA: Deoxyribonucleic acid
CNCI: Coding-non-coding index
PLEK: Predictor of long non-coding RNAs and messenger RNAs based on an improved k-mer scheme
CPAT: Coding potential assessment tool
rRNA: Ribosomal Ribonucleic acid
pfam: Database providing alignments and hidden Markov models for protein domains
mRNA: Messenger Ribonucleic acid
FPKM: Fragments per kilobase of exon per million fragments mapped
CSN2: Casein 2
cDNA: Complementary DNA
DNase: Deoxyribonuclease
dNTP: Deoxyribonucleotide triphosphate
ds: Double-stranded
EDTA: Ethylenediaminetetraacetic acid
Fig: Figure
DE: Differentially expressed
KEGG: Kyoto Encyclopedia of Genes and Genomes
lncRNA: Long non-coding RNA
M: Million
MAPK: Mitogen-activated protein kinase
mRNA: Messenger RNA.
miRNA: MicroRNA
NCBI: National Center for Biotechnology Information
NFκB: Nuclear factor-kappa B
RNA-seq: RNA sequencing
