# Supplementary material for: Mammary epithelial cell transcriptome reveals potential roles of lncRNAs in regulating milk synthesis pathways in Jersey and Kashmiri cattle
Source: BMC Genomics. 2022 Mar 4;23:176. doi: 10.1186/s12864-022-08406-x (PMC8896326; doi:10.1186/s12864-022-08406-x)
Supplement: Supplementary file 1 — Additional file 1. [file 12864_2022_8406_MOESM1_ESM.docx]

| **S.No** | **lncRNA ID** | **Primer Sequence** | **Product Length(bp)** |
| --- | --- | --- | --- |
| 1 | XLOC_009502 | Forward: 5’- CTTAGGGACTCCCCTCGCA-3’ | 70 |
|  |  | Reverse: 5’- AGGGATAGAACCTGTGCCCC-3’ |  |
| 2 | XLOC_002110 | Forward: 5’- CCACTTCTTGCCCCTCCTAT -3’ | 95 |
|  |  | Reverse: 5’- TGCTGTCGGTATTTCTGTGC -3’ |  |
| 3 | XLOC_017603 | Forward: 5’- CTGTTGACGACTGTGGGATG -3’ | 169 |
|  |  | Reverse: 5’- CTCTGGGATTGGGTTAGTGG -3’ |  |
| 4 | XLOC_005053 | Forward: 5’- ATGTTTCACGCCTTCCTCAA -3’ | 99 |
|  |  | Reverse: 5’- CCCGTTTATCCTGTCCTCCT -3’ |  |
| 5 | XLOC_005292 | Forward: 5’- CAAACAAGCCCCACTCTTTC -3’ | 143 |
|  |  | Reverse: 5’- GGAAGCAGAGAGCCACAGAG -3’ |  |
| 6 | XLOC_007175 | Forward: 5’- CTGGGGAAATGAAAAGCAAA -3’ | 97 |
|  |  | Reverse: 5’- TGTGAAGAAAATGGGCATCA -3’ |  |
| 7 | XLOC_020132 | Forward: 5’- CCAAAAATGGGTGAAGTGCT -3’ | 75 |
|  |  | Reverse: 5’- CACTAGGCAACCAGGCAACT -3’ |  |
| 8 | XLOC_002802 | Forward: 5’- TTCAAAGCCAAAAAGGCACT -3’ | 113 |
|  |  | Reverse: 5’- TCCTCCACCGTATTCATTCC -3’ |  |
| 9 | XLOC_003190 | Forward: 5’- CTGGCTGGCTGTGGTCTCTA -3’ | 107 |
|  |  | Reverse: 5’- AACGAAACAAAGATGGCAGAA -3’ |  |
| 10 | XLOC_000504 | Forward: 5’- TTGCCTTCTCATTGCCTTCT -3’ | 96 |
|  |  | Reverse: 5’- AAGTGCTAGAGTGGGGCTCA -3’ |  |
| 11 | GAPDH | Forward: 5’-GCAAGTTCCACGGCACAG -3’ | 249 |
|  |  | Reverse: 5’- GGTTCACGCCCATCACAA -3’ |  |
| 12 | UXT | Forward: 5’- TGGACCATCGTGACAAGGTA -3’ | 155 |
|  |  | Reverse: 5’- TGAAGTGTCTGGGACCACTG -3’ |  |

Supplementary File 1: Primer details of lncRNAs and housekeeping genes used for RT-PCR Validation.
