# Supplementary material for: Mammary epithelial cell transcriptome reveals potential roles of lncRNAs in regulating milk synthesis pathways in Jersey and Kashmiri cattle
Source: BMC Genomics. 2022 Mar 4;23:176. doi: 10.1186/s12864-022-08406-x (PMC8896326; doi:10.1186/s12864-022-08406-x)
Supplement: Supplementary file 4 — Additional file 4. [file 12864_2022_8406_MOESM4_ESM.pdf]

Supplementary File 4: Top gene ontology (GO) terms and pathways enriched for potential target genes of differentially expressed lncRNAs in bovine mammary epithelial cells at different stages of lactation (early lactation (D15), mid lactation (D90) and late lactation (D250) in Jersey (J) and Kashmiri (K) cattle.

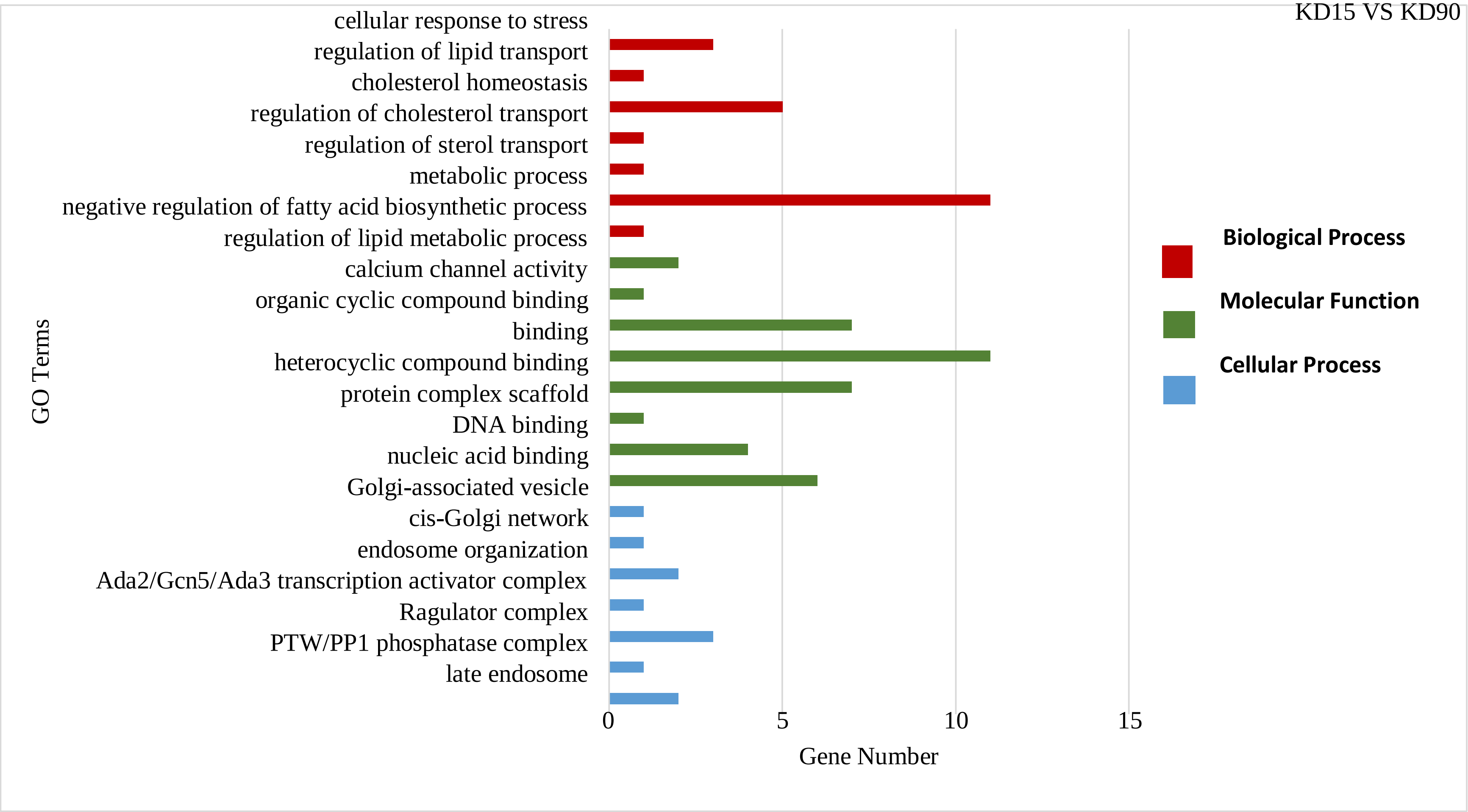
KD15 VS KD90
cellular response to stress
regulation of lipid transport
cholesterol homeostasis
regulation of cholesterol transport
regulation of sterol transport
metabolic process
negative regulation of fatty acid biosynthetic process
regulation of lipid metabolic process
calcium channel activity
organic cyclic compound binding
binding
heterocyclic compound binding
protein complex scaffold
DNA binding
nucleic acid binding
Golgi-associated vesicle
cis-Golgi network
endosome organization
Ada2/Gcn5/Ada3 transcription activator complex
Ragulator complex
PTW/PP1 phosphatase complex
late endosome
GO Terms
0
5
10
15
Gene Number
Biological Process
Molecular Function
Cellular Process

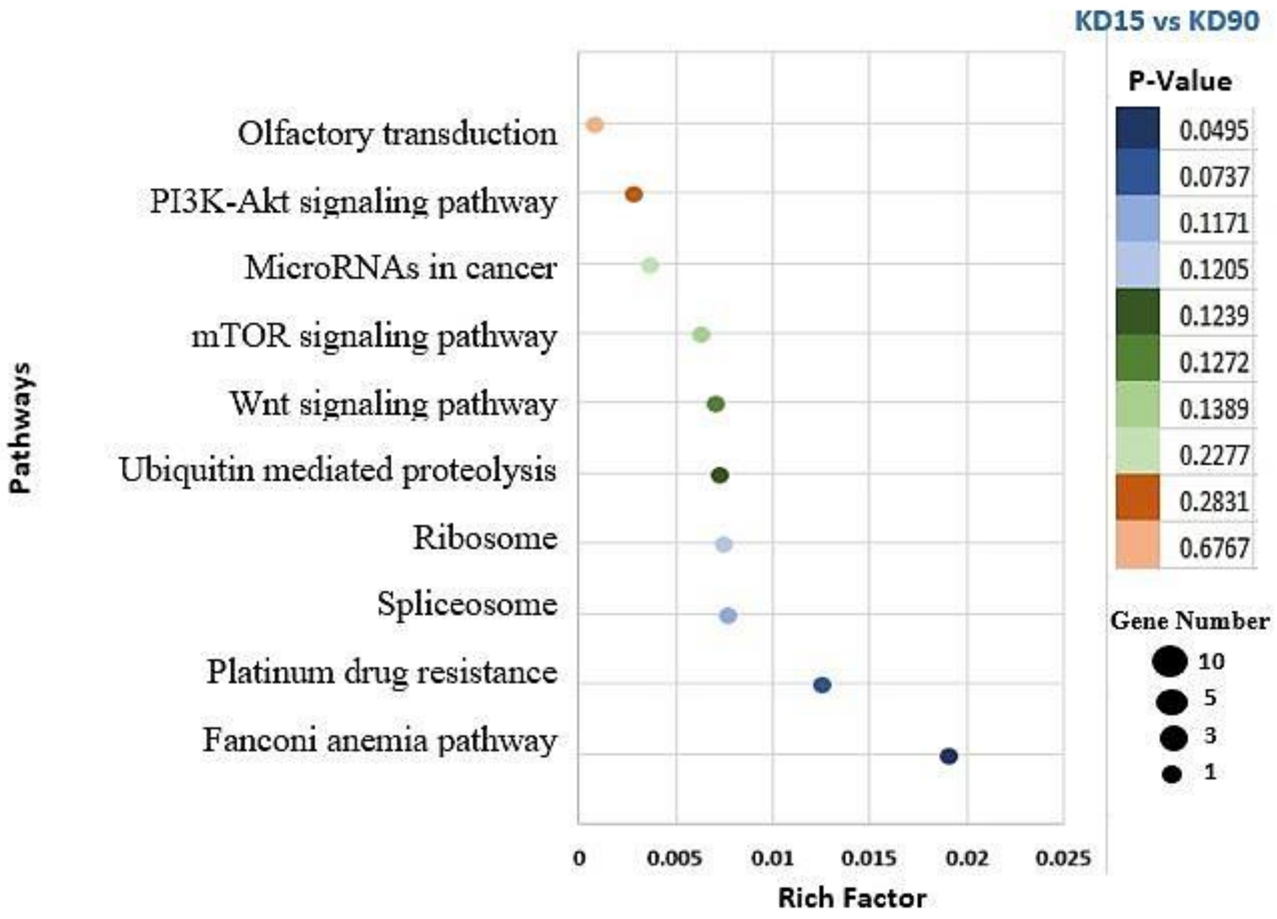
KD15 vs KD90
P-Value
0.0495
0.0737
0.1171
0.1205
0.1239
0.1272
0.1389
0.2277
0.2831
0.6767
Gene Number
10
5
3
1
Pathways
Olfactory transduction
PI3K-Akt signaling pathway
MicroRNAs in cancer
mTOR signaling pathway
Wnt signaling pathway
Ubiquitin mediated proteolysis
Ribosome
Spliceosome
Platinum drug resistance
Fanconi anemia pathway
0
0.005
0.01
0.015
0.02
0.025
Rich Factor

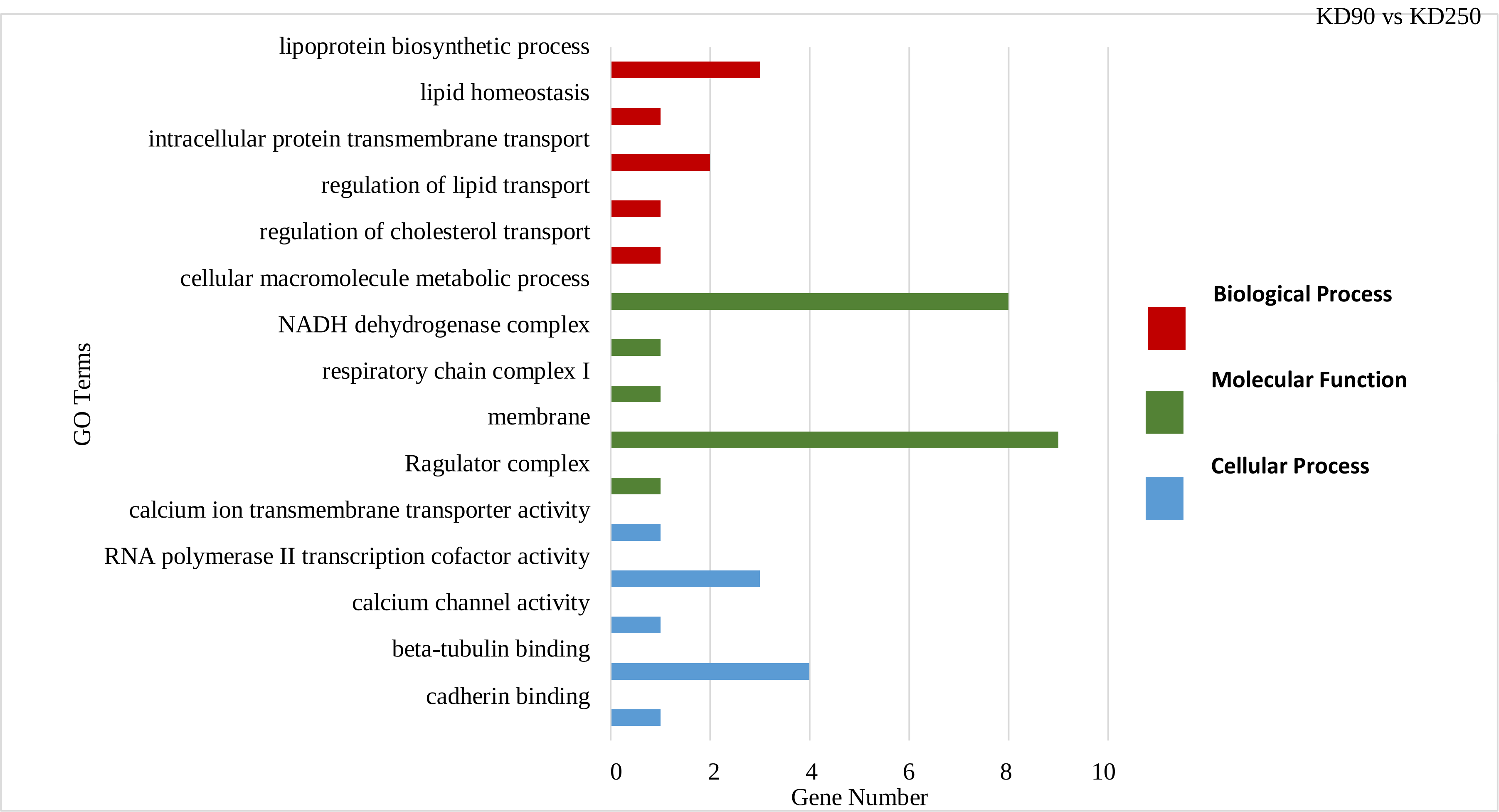
KD90 vs KD250
GO Terms
lipoprotein biosynthetic process
lipid homeostasis
intracellular protein transmembrane transport
regulation of lipid transport
regulation of cholesterol transport
cellular macromolecule metabolic process
NADH dehydrogenase complex
respiratory chain complex I
membrane
Ragulator complex
calcium ion transmembrane transporter activity
RNA polymerase II transcription cofactor activity
calcium channel activity
beta-tubulin binding
cadherin binding
0
2
4
6
8
10
Gene Number
Biological Process
Molecular Function
Cellular Process

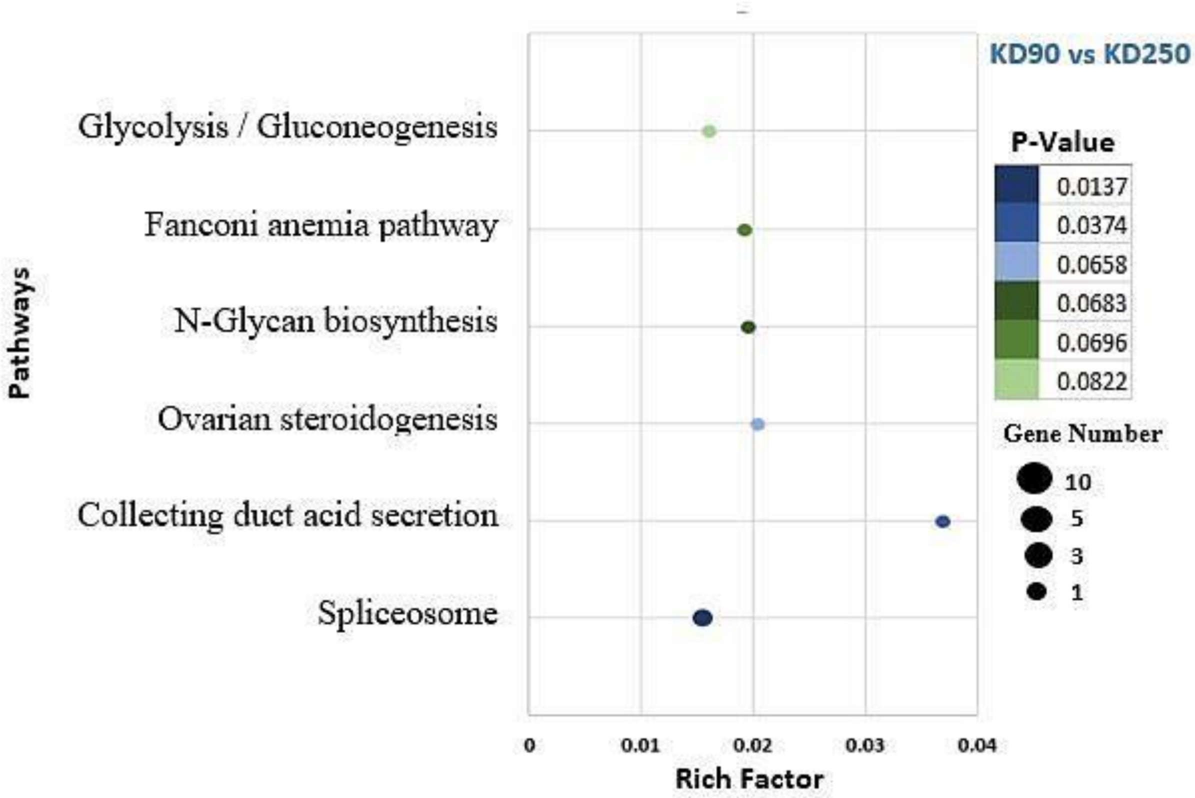

KD90 vs KD250
Glycolysis / Gluconeogenesis
Fanconi anemia pathway
N-Glycan biosynthesis
Ovarian steroidogenesis
Collecting duct acid secretion
Spliceosome
Pathways
P-Value
0.0137
0.0374
0.0658
0.0683
0.0696
0.0822
Gene Number
10
5
3
1
0
0.01
0.02
0.03
0.04
Rich Factor

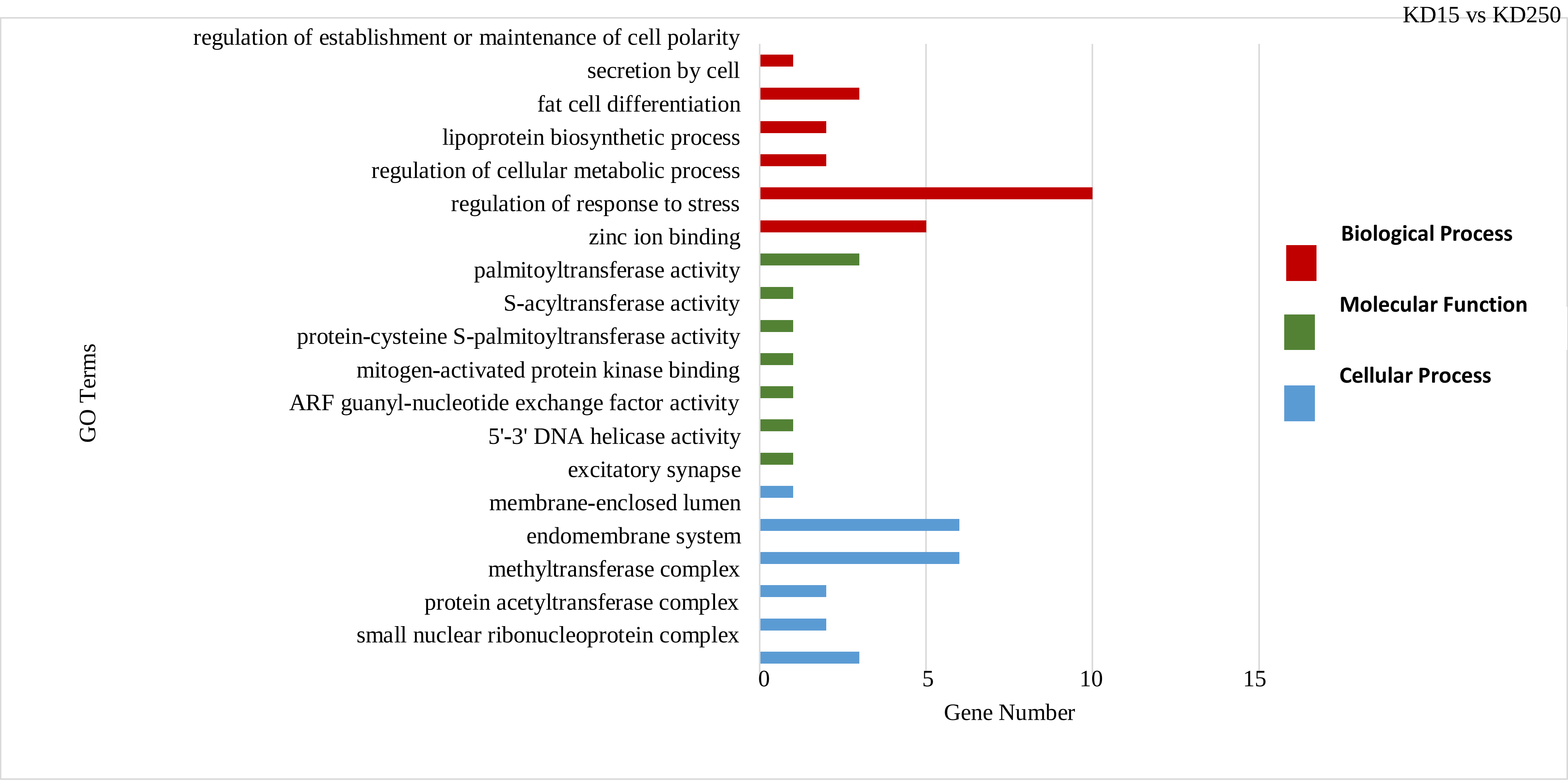
KD15 vs KD250
GO Terms
regulation of establishment or maintenance of cell polarity
secretion by cell
fat cell differentiation
lipoprotein biosynthetic process
regulation of cellular metabolic process
regulation of response to stress
zinc ion binding
palmitoyltransferase activity
S-acyltransferase activity
protein-cysteine S-palmitoyltransferase activity
mitogen-activated protein kinase binding
ARF guanyl-nucleotide exchange factor activity
5'-3' DNA helicase activity
excitatory synapse
membrane-enclosed lumen
endomembrane system
methyltransferase complex
protein acetyltransferase complex
small nuclear ribonucleoprotein complex
0
5
10
15
Gene Number
Biological Process
Molecular Function
Cellular Process

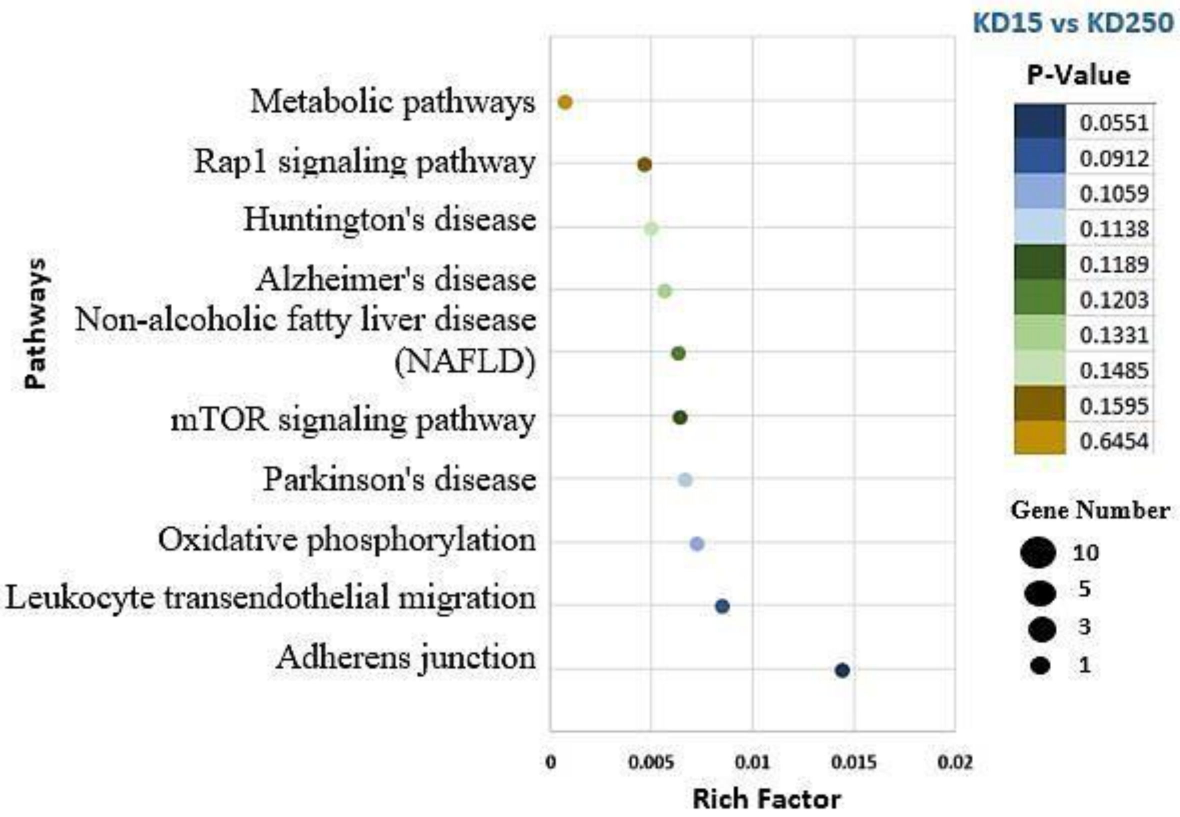

KD15 vs KD250
P-Value
0.0551
0.0912
0.1059
0.1138
0.1189
0.1203
0.1331
0.1485
0.1595
0.6454
Gene Number
10
5
3
1
Pathways
Metabolic pathways
Rap1 signaling pathway
Huntington's disease
Alzheimer's disease
Non-alcoholic fatty liver disease (NAFLD)
mTOR signaling pathway
Parkinson's disease
Oxidative phosphorylation
Leukocyte transendothelial migration
Adherens junction
0
0.005
0.01
0.015
0.02
Rich Factor

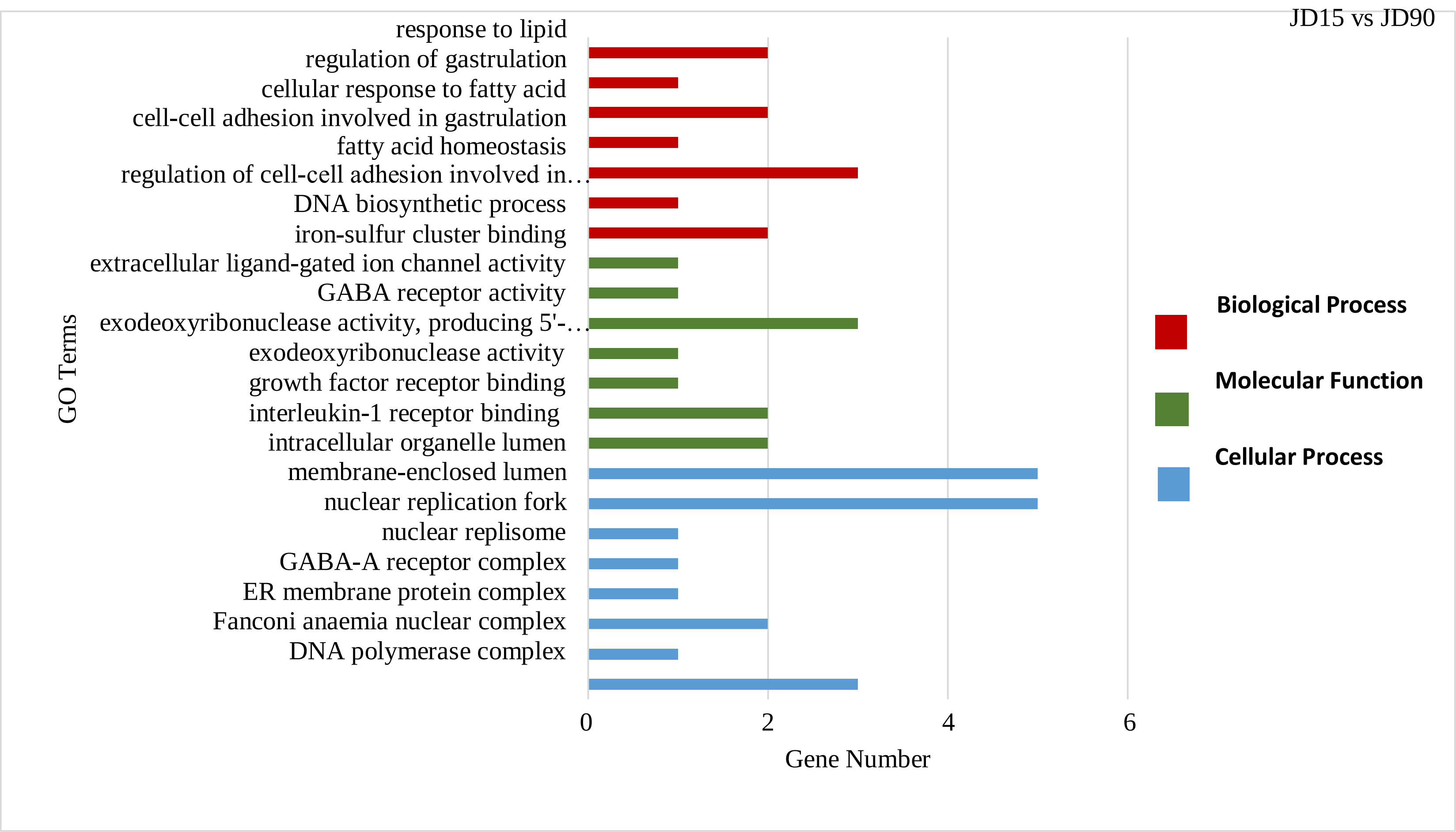
JD15 vs JD90
GO Terms
response to lipid
regulation of gastrulation
cellular response to fatty acid
cell-cell adhesion involved in gastrulation
fatty acid homeostasis
regulation of cell-cell adhesion involved in…
DNA biosynthetic process
iron-sulfur cluster binding
extracellular ligand-gated ion channel activity
GABA receptor activity
exodeoxyribonuclease activity, producing 5'-…
exodeoxyribonuclease activity
growth factor receptor binding
interleukin-1 receptor binding
intracellular organelle lumen
membrane-enclosed lumen
nuclear replication fork
nuclear replisome
GABA-A receptor complex
ER membrane protein complex
Fanconi anaemia nuclear complex
DNA polymerase complex
0
2
4
6
Gene Number
Biological Process
Molecular Function
Cellular Process

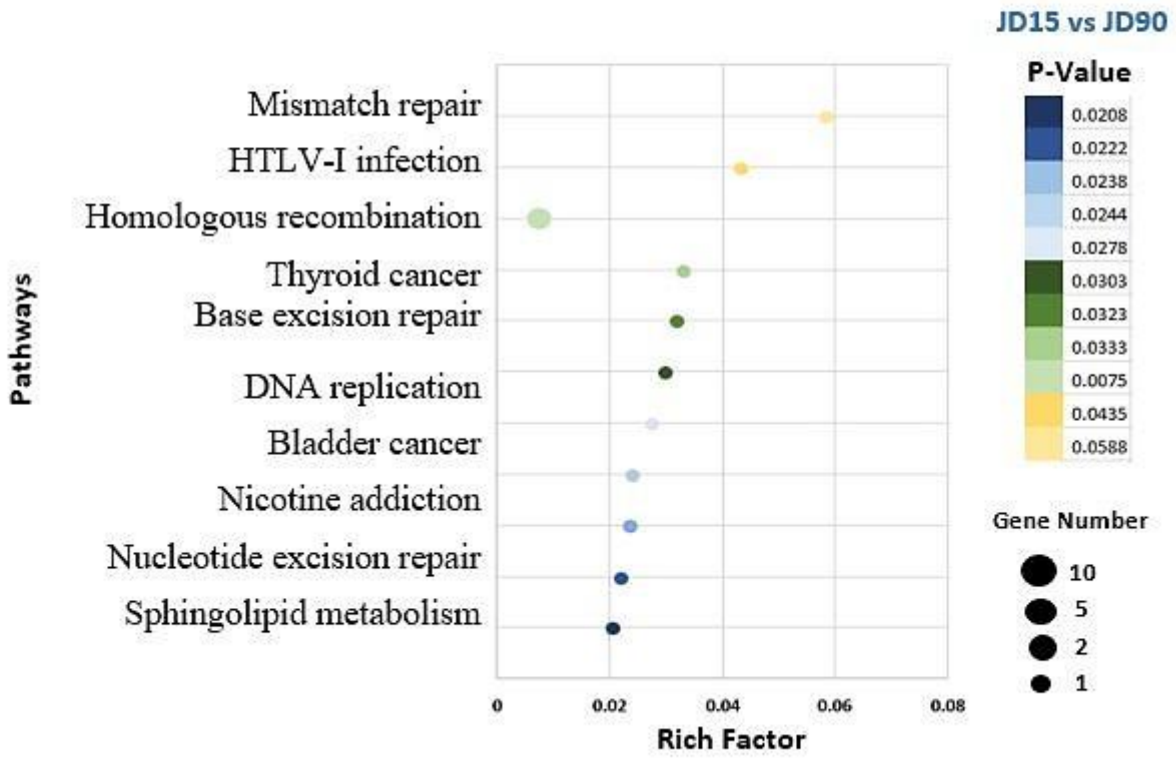

JD15 vs JD90
P-Value
0.0208
0.0222
0.0238
0.0244
0.0278
0.0303
0.0323
0.0333
0.0075
0.0435
0.0588
Gene Number
10
5
2
1
Mismatch repair
HTLV-I infection
Homologous recombination
Thyroid cancer
Base excision repair
DNA replication
Bladder cancer
Nicotine addiction
Nucleotide excision repair
Sphingolipid metabolism
Pathways
0
0.02
0.04
0.06
0.08
Rich Factor

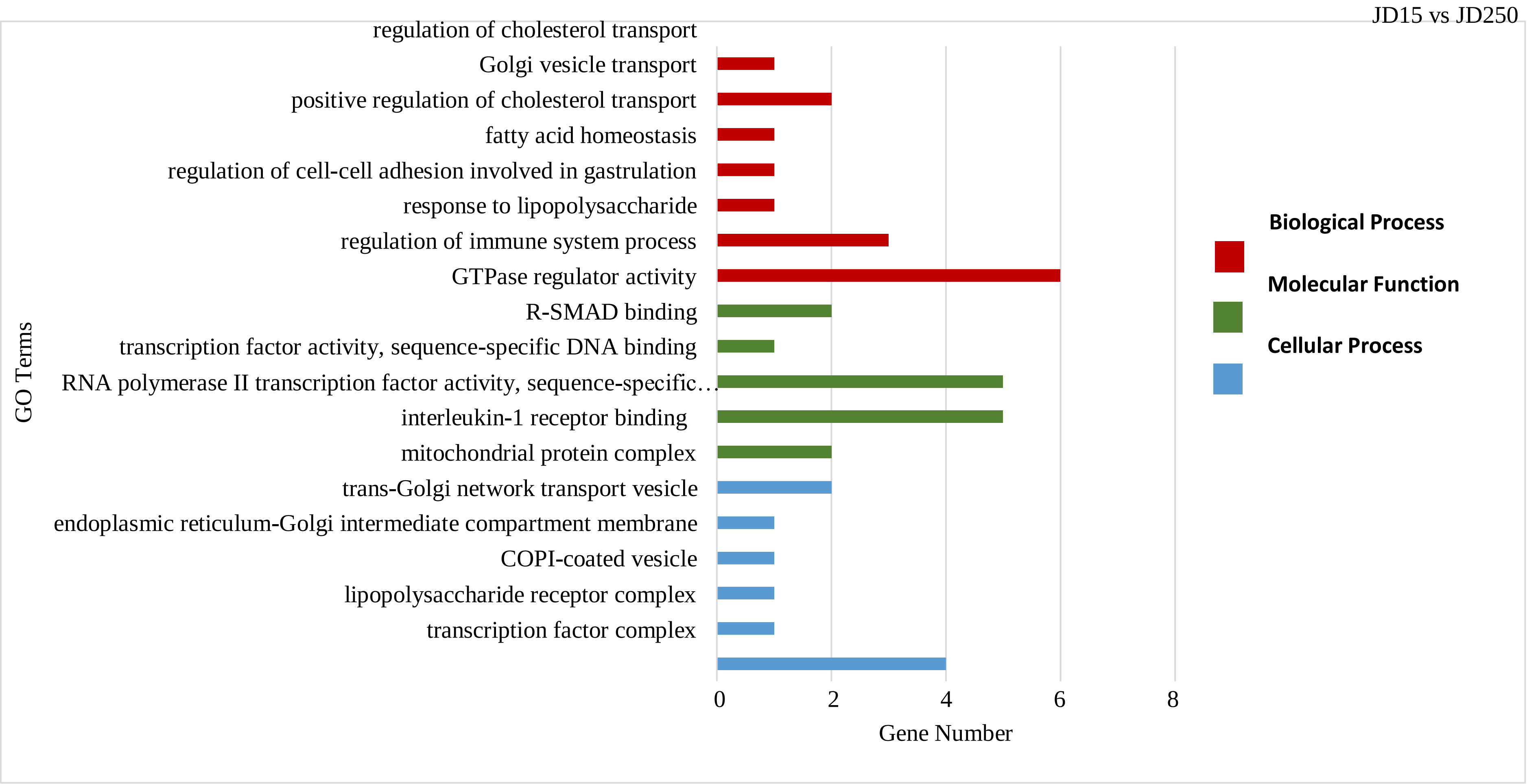
JD15 vs JD250
regulation of cholesterol transport
Golgi vesicle transport
positive regulation of cholesterol transport
fatty acid homeostasis
regulation of cell-cell adhesion involved in gastrulation
response to lipopolysaccharide
regulation of immune system process
GTPase regulator activity
R-SMAD binding
transcription factor activity, sequence-specific DNA binding
RNA polymerase II transcription factor activity, sequence-specific…
interleukin-1 receptor binding
mitochondrial protein complex
trans-Golgi network transport vesicle
endoplasmic reticulum-Golgi intermediate compartment membrane
COPI-coated vesicle
lipopolysaccharide receptor complex
transcription factor complex
GO Terms
0
2
4
6
8
Gene Number
Biological Process
Molecular Function
Cellular Process

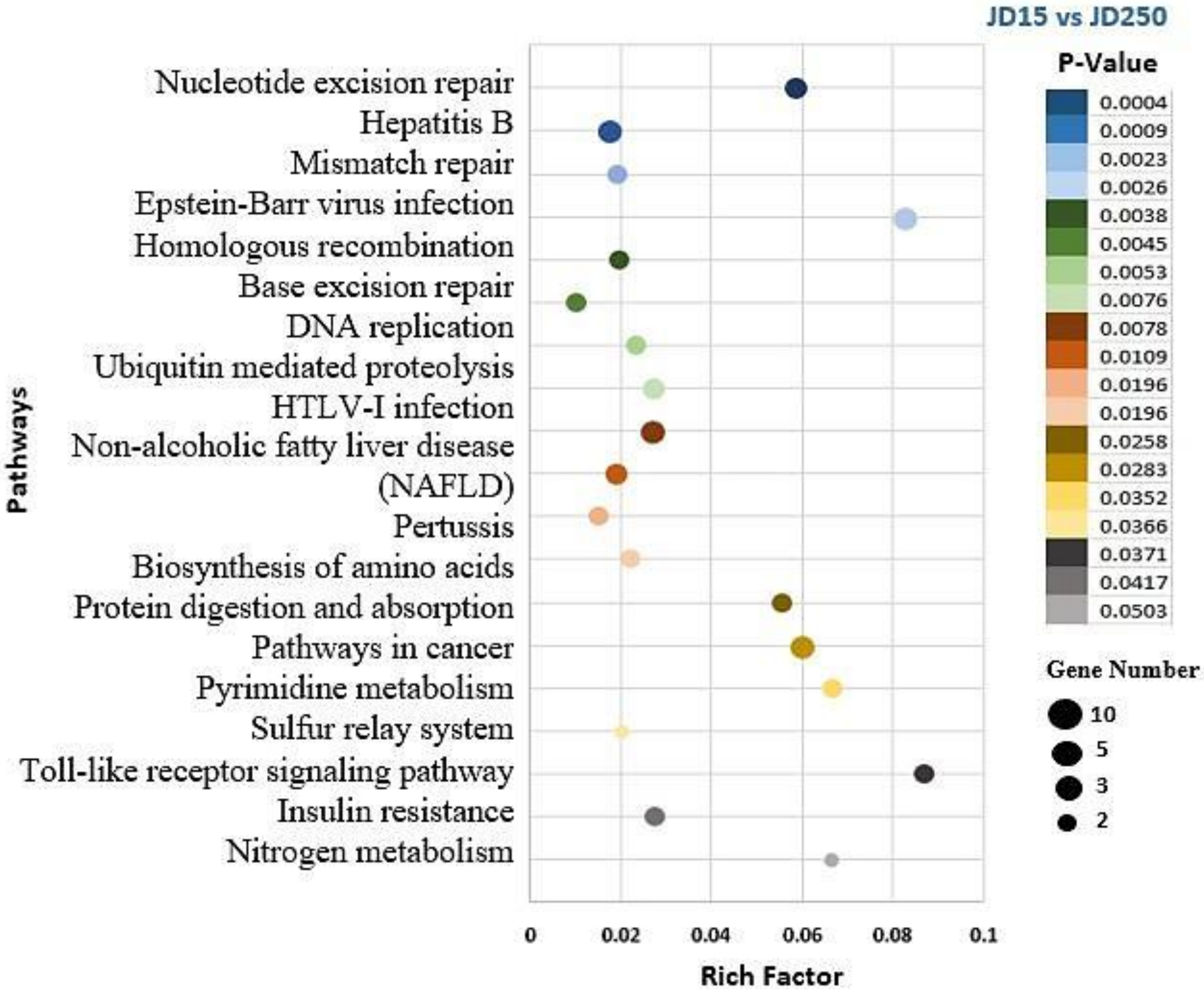

JD15 vs JD250
P-Value
0.0004
0.0009
0.0023
0.0026
0.0038
0.0045
0.0053
0.0076
0.0078
0.0109
0.0196
0.0196
0.0258
0.0283
0.0352
0.0366
0.0371
0.0417
0.0503
Gene Number
10
5
3
2
Pathways
Nucleotide excision repair
Hepatitis B
Mismatch repair
Epstein-Barr virus infection
Homologous recombination
Base excision repair
DNA replication
Ubiquitin mediated proteolysis
HTLV-I infection
Non-alcoholic fatty liver disease (NAFLD)
Pertussis
Biosynthesis of amino acids
Protein digestion and absorption
Pathways in cancer
Pyrimidine metabolism
Sulfur relay system
Toll-like receptor signaling pathway
Insulin resistance
Nitrogen metabolism
0
0.02
0.04
0.06
0.08
0.1
Rich Factor

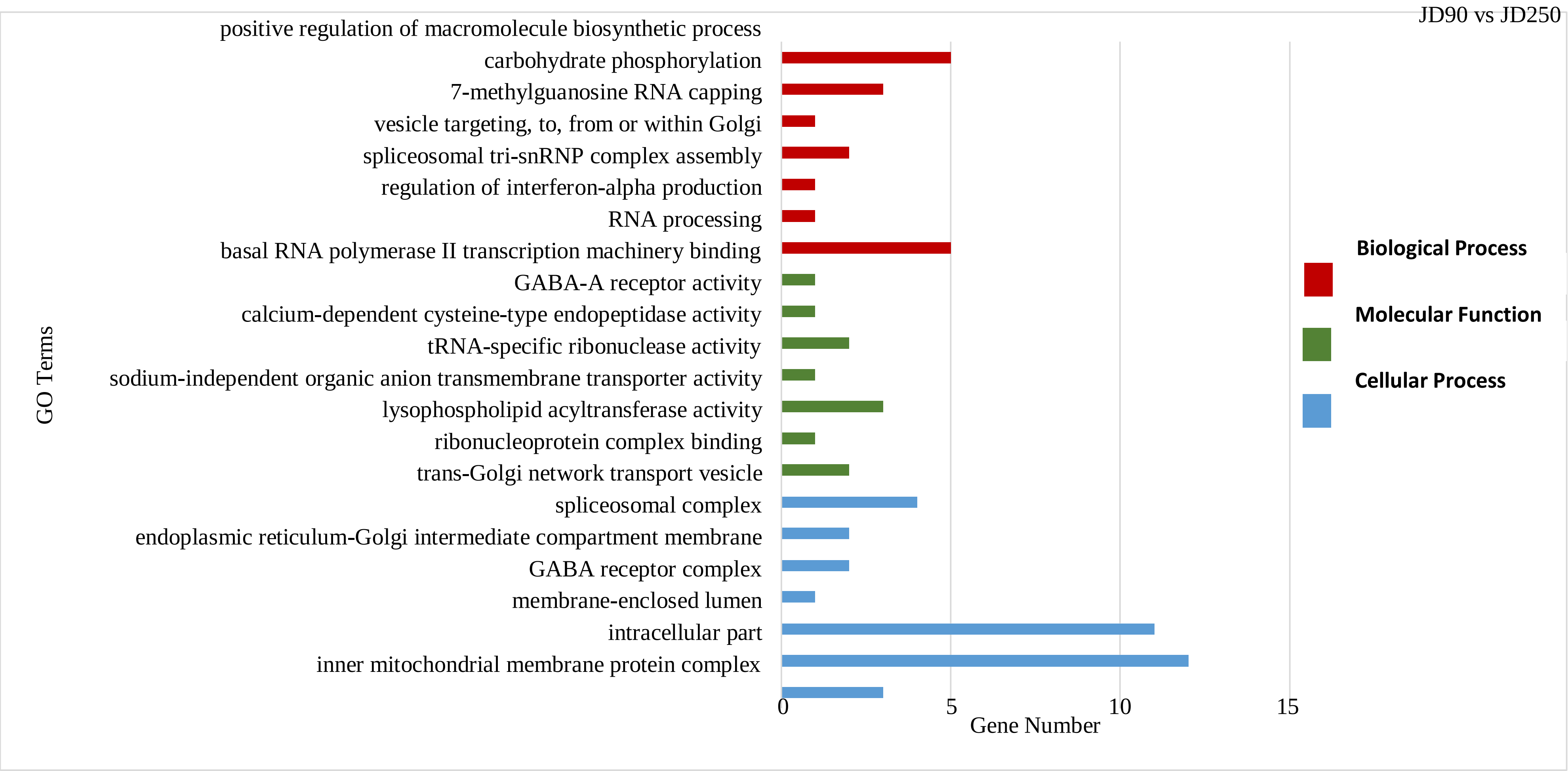

JD90 vs JD250
positive regulation of macromolecule biosynthetic process
carbohydrate phosphorylation
7-methylguanosine RNA capping
vesicle targeting, to, from or within Golgi
spliceosomal tri-snRNP complex assembly
regulation of interferon-alpha production
RNA processing
basal RNA polymerase II transcription machinery binding
GABA-A receptor activity
calcium-dependent cysteine-type endopeptidase activity
tRNA-specific ribonuclease activity
sodium-independent organic anion transmembrane transporter activity
lysophospholipid acyltransferase activity
ribonucleoprotein complex binding
trans-Golgi network transport vesicle
spliceosomal complex
endoplasmic reticulum-Golgi intermediate compartment membrane
GABA receptor complex
membrane-enclosed lumen
intracellular part
inner mitochondrial membrane protein complex
GO Terms
0
5
10
15
Gene Number
Biological Process
Molecular Function
Cellular Process

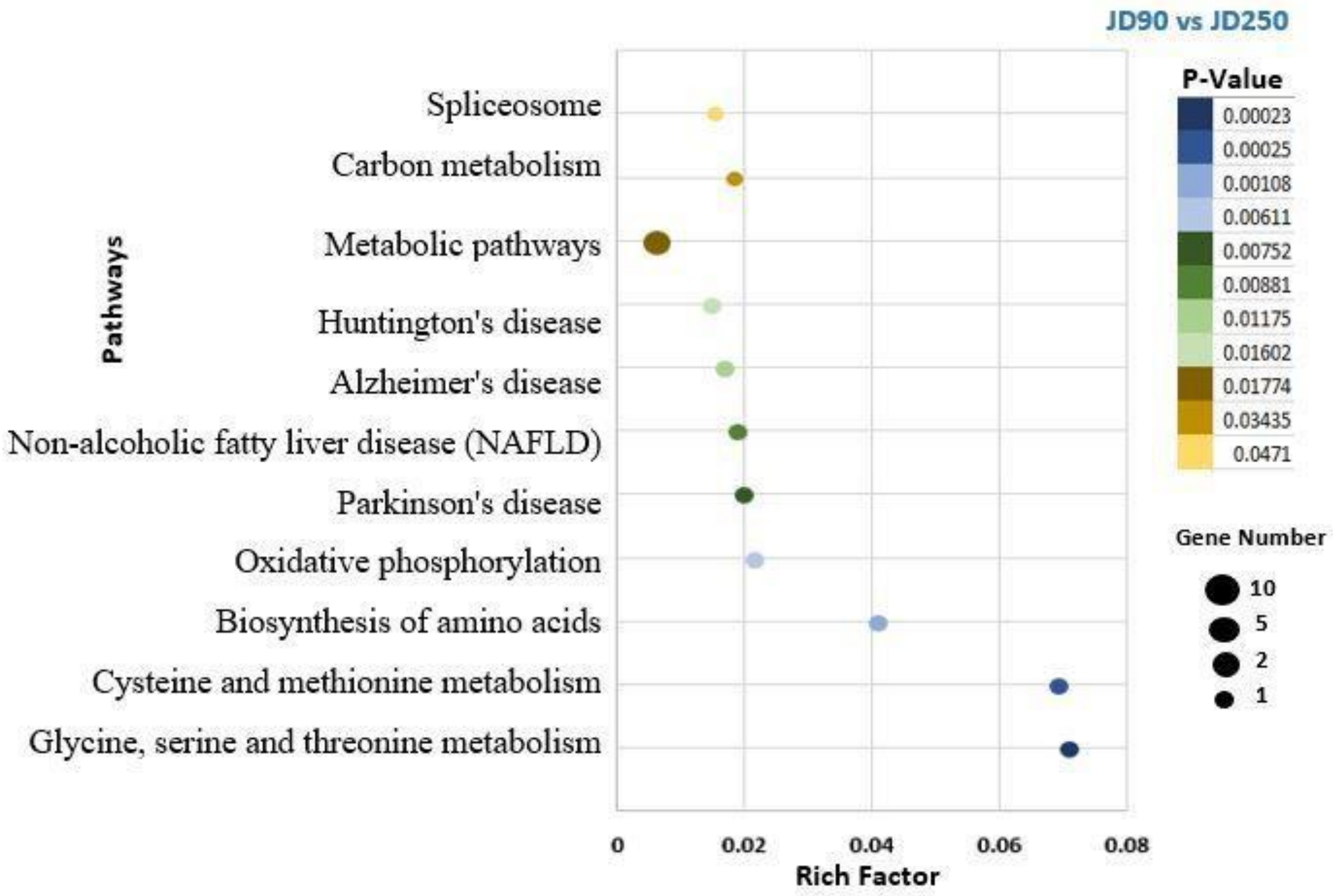
JD90 vs JD250
Pathways
Spliceosome
Carbon metabolism
Metabolic pathways
Huntington's disease
Alzheimer's disease
Non-alcoholic fatty liver disease (NAFLD)
Parkinson's disease
Oxidative phosphorylation
Biosynthesis of amino acids
Cysteine and methionine metabolism
Glycine, serine and threonine metabolism
0
0.02
0.04
0.06
0.08
Rich Factor
P-Value
0.00023
0.00025
0.00108
0.00611
0.00752
0.00881
0.01175
0.01602
0.01774
0.03435
0.0471
Gene Number
10
5
2
1
